# Anatomy and connectivity of the Göttingen minipig subgenual cortex (Brodmann area 25 homologue)

**DOI:** 10.1007/s00429-024-02855-8

**Published:** 2024-09-28

**Authors:** Andreas N. Glud, Hamed Zaer, Dariusz Orlowski, Mette Slot Nielsen, Jens Christian H. Sørensen, Carsten R. Bjarkam

**Affiliations:** 1 Department of Neurosurgery, Center for Experimental Neuroscience (CENSE), Aarhus University Hospital, 8200 Aarhus N, Denmark; 2 Department of Neurosurgery, Aalborg University Hospital, Aalborg, Denmark

**Keywords:** Brodmann area 25, Cytoarchitecture, Depression, Neuronal tracing, Sus scrofa, Translational anatomy

## Abstract

**Background:**

The subgenual gyrus is a promising target for deep brain stimulation (DBS) against depression. However, to optimize this treatment modality, we need translational animal models.

**Aim:**

To describe the anatomy and connectivity of the Göttingen minipig subgenual area (sgC).

**Materials and methods:**

The frontal pole of 5 minipigs was cryosectioned into 40 μm coronal and horizontal sections and stained with Nissl and NeuN-immunohistochemistry to visualize cytoarchitecture and cortical lamination. Eight animals were unilaterally stereotaxically injected in the sgC with anterograde (BDA) and retrograde (FluoroGold) tracers to reveal the sgC connectivity.

**Results:**

In homology with human nomenclature (Brodmann 1909), the minipig sgC can be subdivided into three distinct areas named area 25 (BA25), area 33 (BA33), and indusium griseum (IG). BA25 is a thin agranular cortex, approximately 1 mm thick. Characteristically, perpendicular to the pial surface, cell-poor cortical columns separate the otherwise cell-rich cortex of layer II, III and V. In layer V the cells are of similar size as seen in layer III, while layer VI contains more widely dispersed neurons. BA33 is less differentiated than BA25. Accordingly, the cortex is thinner and displays a complete lack of laminar differentiation due to diffusely arranged small, lightly stained neurons. It abuts the IG, which is a neuron-dense band of heavily stained small neurons separating BA33 directly from the corpus callosum and the posteriorly located septal nuclear area.

Due to the limited area size and nearby location to the lateral ventricle and longitudinal cerebral fissure, only 3/8 animals received sgC injections with an antero- and retrograde tracer mixture. Retrograde tracing was seen primarily to the neighbouring ipsilateral ventral- and mPFC areas with some contralateral labelling as well. Prominent projections were furthermore observed from the ipsilateral insula, the medial aspect of the amygdala and the hippocampal formation, diencephalon and the brainstem ventral tegmental area. Anterograde tracing revealed prominent projections to the neighbouring medial prefrontal, mPFC and cingulate cortex, while moderate staining was noted in the hippocampus and adjoining piriform cortex.

**Conclusion:**

The minipig sgC displays a cytoarchitectonic pattern and connectivity like the human and may be well suited for further translational studies on BA25-DBS against depression.

## Introduction

Humans suffering of treatment resistant depression may display morphological and functional changes in prefrontal areas and circuitry (Drevets et al. 1997; Manji et al. 2001; Botteron et al.^2002^) Accordingly, deep brain stimulation of the prefrontal BA25 area (BA25-DBS) has been introduced to treat medically refractory depression (Mayberg et al. 2005; Lozano et al. 2012; Holtzheimer et al. 2017; Breen et al. 2018; Sankar et al. 2020).

Previous studies have described the comparative anatomy of BA25, evaluating its connections with neuronal tracing in order to explore the potential of rodent prelimbic homologue models (Sharma et al. 2020). The interest in BA25 and its role in emotional processing, evaluation of fear and depression through amygdalar inputs, connections of the dorsal, perigenual, and subgenual anterior cingulate cortices (Kim et al. 2018) have paved the way for translational large animal modeling of fear and depression. With connections modulating both arousal and conflict monitoring, the functional anatomical region adds to the complexity of both basic scientific questions and clinical behavioral treatments (Kelly et al. 2021).

The Göttingen minipig (Crossbreed of the Minnesota mini-pig, the Vietnamese Pot-bellied pig, and the German Landrace pig, *Sus scrofa domesticus*) may be well suited for such translational studies as we previously have developed stereotaxic equipment and techniques (Bjarkam et al. 2004, 2008a, b, 2009; Ettrup et al. 2011b) enabling high precision implantation of deep brain stimulation electrodes in the Göttingen minipig hypothalamus, (Ettrup et al. 2012), striatum (Orlowski et al. 2017) and subthalamic nucleus (Christensen et al. 2018) for subsequent studies based on brain imaging with PET and MRI, and postmortem histopathological analysis (Sørensen et al. 2000; Bjarkam et al. 2001; Larsen et al. 2004; Bjarkam et al. 2004; Bjarkam et al. 2008b; Bjarkam et al. 2008a; Rosendal et al. 2009b; Nielsen et al. 2009; Bjarkam et al. 2009a; Rosendal et al. 2009a; Bjarkam et al. 2010; Nørgaard Glud et al. 2010; Ettrup et al. 2010; Fjord-Larsen et al. 2010; Glud et al. 2011a; Ettrup et al. 2011b; Ettrup et al. 2012; Meidahl et al. 2016a; Nielsen et al. 2016; Glud et al. 2016; Bjarkam et al. 2017b; Glud et al. 2017a; Bjarkam et al. 2017a; Orlowski et al. 2017; Christensen et al. 2018; Lillethorup et al. 2018a; Lillethorup et al. 2018c; Bech et al. 2018; Lillethorup et al. 2018b; Orlowski et al. 2019; Glud et al. 2019; Bech et al. 2020; Zaer et al. 2020; Tvilling et al. 2021). However, more fundamental knowledge on the Göttingen minipig BA25 anatomy is needed before a successful DBS model for depression can be made.

## Aim

To describe the anatomy and connectivity of the Göttingen minipig sgC and compare these findings to the known anatomy of the human, non-human primate, and rodent BA25 area in order to establish the anatomical foundation for a Göttingen minipig model of BA25-DBS.

## Materials and methods

### Animals

13 female Göttingen minipigs 6–12 months old weighing 20–25 kg were used in this study in accordance with a protocol approved by the Danish Animal Expectorate (journal number 2003-561-705). Female animals were used as males are: larger, more territorial, must be housed separate from females, and have a special anatomy that does not allow urine catheters during surgery.

### Anatomy study

#### Tissue preparation and handling

Animals were transcardial perfused using 5 L buffered formalin (VWR Bie & Berntsen, Søborg, Denmark), as previously described (Ettrup et al 2011a).

Skull was opened by a using surgical hammer and chisel in order to expose the dura. Dura was then opened using scissors and forceps before the brain could be removed *in toto* as previously described (Bjarkam et al. 2017a, 2017b). After removal the brains were post-fixed it the same fixative for one week and cut in 20 mm coronal tissue blocks using the HistOtech^®^ slicer (Bjarkam et al. 2001). After 8–10 days in 30% sucrose the tissue blocks were frozen as described above and sectioned into 40 μm coronal sections on a cryostat. (Ettrup et al. 2010).

#### Histo- and immunohistochemistry procedures

Mounted frontal and horizontal sections were stained with Nissl to visualize neurons and glial cells. The staining procedure was as follows: sections were stained with 0.1% toluidine- blue in citrate buffer (pH 4.0) at room temperature for 4 min, followed by 2 × rinsing in distilled water, 3 × dehydration in 99% alcohol, submerged in xylene and coverslipped with Depex.

The immunohistochemical procedures were performed according to the avidin–biotin method (Bjarkam et al. 2005). The antibody used was a monoclonal antibody directed against the neuron-specific nuclear protein (NeuN) (Mouse Anti- neuronal nuclei (NeuN) monoclonal antibody, cat no MAB377, Chemicon International Inc, Harrow, UK). Free-floating coronal and horizontal sections stored in cryoprotection fluid were selected for immunohistochemistry. Free-floating sections from one series were initially rinsed twice in Tris-buffered saline (TBS; 0.05 M; pH 7.4) for 15 min. and then once in TBS plus 1% Triton X-100 (TBS-T) for 10 min. The endogenous peroxidase was blocked by washing sections in TBS containing 3% H_2_O_2_ and 10% methanol two times for 15 min. following by rinsing in TBS-T for 3 × 5 min. Pre-incubation with TBS-T and 0.2% milk (Bidinger, Aarhus, Denmark) was performed for 30 min. prior to incubation with the primary antibody (NeuN antibody diluted 1:500 in 1% Triton X-100 and 0.2% milk) for 72 h at 4 °C. Hereafter, the sections were rinsed for 3 × 10 min. in TBS-T both before and next after the incubation with the secondary antibody (sheep, anti-mouse Ig antibody (Amersham RPN 1001) diluted 1:200 in TBS and 1% Triton X- 100 and 0.2% milk) for 1 h at room temperature. The sections were then rinsed in TBS and 1% Triton X- 100 for 3 × 15 min and incubated with avidin-peroxidase (Sigma; A 3151) diluted 1:200 in TBS containing 1% Triton X-100 and 0.2% milk for 1 h at room temperature. After rinsing for 3 × 15 min in TBS + 1% Triton X-100 the avidin-peroxidase complex was visualized by incubation for approximately 10 min with diaminobenzidine (DAB). The reaction was visualized using 0.1% DAB (3,3 Diaminobenzidin-tetrachloride dehydrate, cat.no.: 4170, Kem-En-Tec Diagnostics A/S, DK) with 0.3% H2O2. Primary (mouse anti-NeuN) RRID: AB_2298772 Secondary (sheep anti-mouse) RRID: AB_1062579.

The visualization process was stopped by moving the sections to distilled water followed by mounting on slides, air drying and dehydration in increasing alcohol solutions, alcohol, submerged in xylene and cover slipped with Depex. Negative control procedures omitting the primary antibody were performed.

#### Tracing study

Eight female Göttingen minipigs received a unilateral tracer injection containing the primarily anterograde fluorescent tracer dextran tetramethylrhodamine + biotin (BDA-rhod) (Mini-ruby, 10 kDa, cat no D3312, Molecular Probes Inc., Eugene, OR) (Reiner and Honig, 2006; Reiner et al., 2000; Schmued and Fallon 1986; Veenman et al., 1992) and the selectively fluorescent retrograde tracer hydroxystilbamidine methanesulfonate (FG) (equivalent to the retrograde tracer FluoroGold from Fluorochrome, Denver, CO) (Cat no H22845, Molecular Probes Inc., Eugene, OR) (Schmued and Fallon 1986; Ju et al. 1989; Catapano et al. 2008) diluted to 5% and 2% in double-distilled water, respectively.

### Surgery procedure

Animals were sedated (Mix of Midazolam i.m. and Fentanyl i.m. according to weight) before intubation and mechanical gas ventilation through the entire surgery. Tracer targets were planed using a MR-compatible stereotaxic localizor box, that allows targeting of cortical and subcortical structures with a human intended surgical operation computer (Surgiplan, Leksell). The animals were MR-scanned using a 3 T Siemens Skyra. The skin was opened linear in the midline using a scalpel, and the revealed skull was opened using a human intended electric drill (Midas Rex). Dura was gently opened with a small scalpel. (Bjarkam et al. 2009b; Ettrup et al. 2011a; Glud et al. 2011b; Meidahl et al. 2016b; Glud et al. 2017b).

A 1μL Hamilton microsyringe was stereotaxically guided, via Microdrive (TSE), into the estimated target followed by injection of 0.3 μL 5% BDA- rhod and 2% hydroxystilbamidine solution (Sørensen et al. 1995).

Postoperative care included intramuscular injection of 1 mL Buprenorphine (Temgesic Buprenorphinum, 0.3 mg/mL, vnr. no. 060422, Schering-Plough) and antibiotics (Cefuroxim Stragen, 750 mg, vnr.no. 435750, Stragen Nordic) for three days. All operating procedures were carried out under aseptic conditions utilizing sterile utensils and covers. The animals were monitored during the surgical procedures by controlling the blood oxygen saturation, pulse, non-invasive blood pressure, and rectal temperature.

### Tissue preparation and handling

To ensure that the tracers will reach the target structures, the animals were sacrificed after 14 days from the surgery (Bech et al. 2018).

### BDA processing

Samples were snap-frozen before sectioning into 40 μm thick sections using a cryostat. The BDA-rho tracer binds biotin, making it possible to obtain permanent labelling. The visualization process is the same as described under the immunohistochemical procedure omitting the primary and secondary antibody steps and starting with endogenous tissue peroxidase inactivation.

## Results

### Midsagittal surface anatomy

**Fig. 1:**
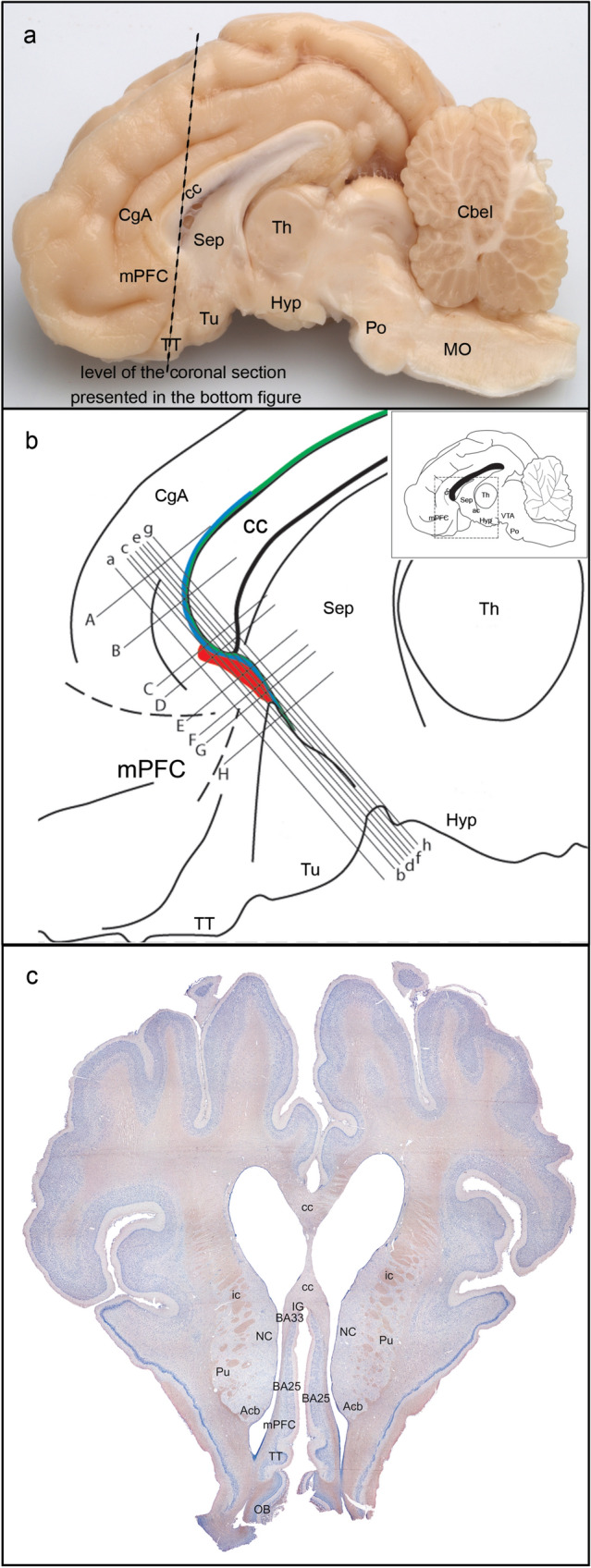
**a** Midsagittal section through the minipig brain displaying the corpus callosum (cc) and related anterior cingulate gyrus (CgA), which ventrally continue into the sgC at the transition zone between the mPFC, the septal nuclear area (Sep) and the olfactory tubercle (Tu). **b** Enlarged schematic drawing illustrating the location and subsegregation of the sgC into area 25 (red), area 33 (blue), and IG (green). **C** Overview of the coronal plane with the areas of interest. Lines a-h indicates the level of the coronal sections displayed in Fig. 3, whereas lines A-H indicates the level of the horizontal sections displayed in Fig. 4

Figure 1 depicts the major subdivisions of the Göttingen minipig brain seen on a midsagittal section. The anterior cingulate gyrus CgA curves anteriorly around the genu of corpus callosum (cc) where it ventrally and slightly posteriorly gradually is replaced by the subgenual cortical area (sgC) (Fig. 1B), which posteriorly is demarcated by the septal nuclear area and more ventrally by the olfactory tubercle and the tenia tecta (Bjarkam et al. 2017a). The sgC is anteriorly demarcated by more clearly laminated and thicker parts of the medial prefrontal cortex (mPFC). Still, there is no macroscopic structure that would clearly mark this transition.

### The Göttingen minipig subgenual cortical area (sgC)

**Fig. 2:**
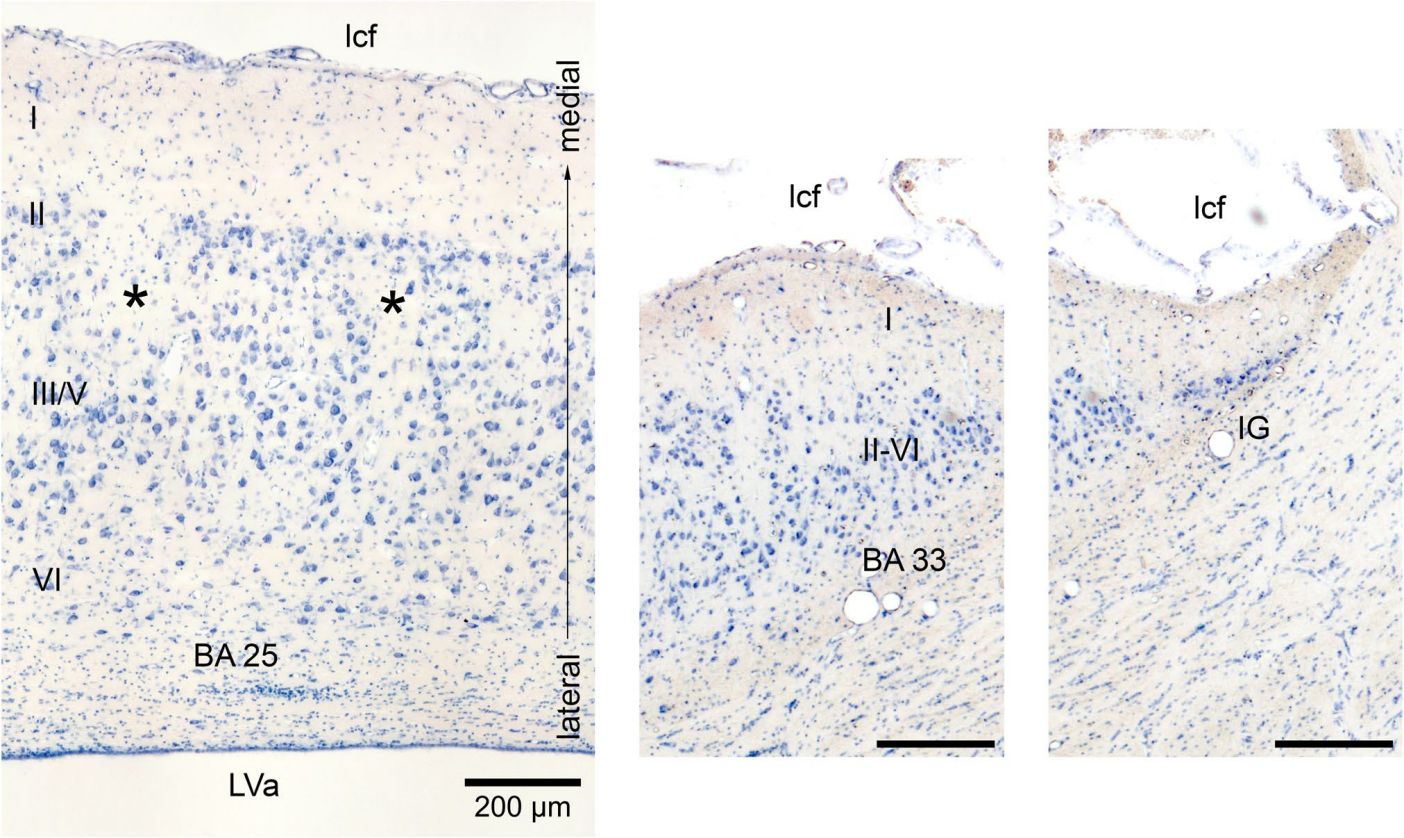
Microphotographs of selected parts of the Nissl-stained coronal section in Fig. 1C, depicting the cytoarchitecture of the BA25, BA33 and Indusium griseum areas. Scale bar 200 μm

**Fig. 3:**
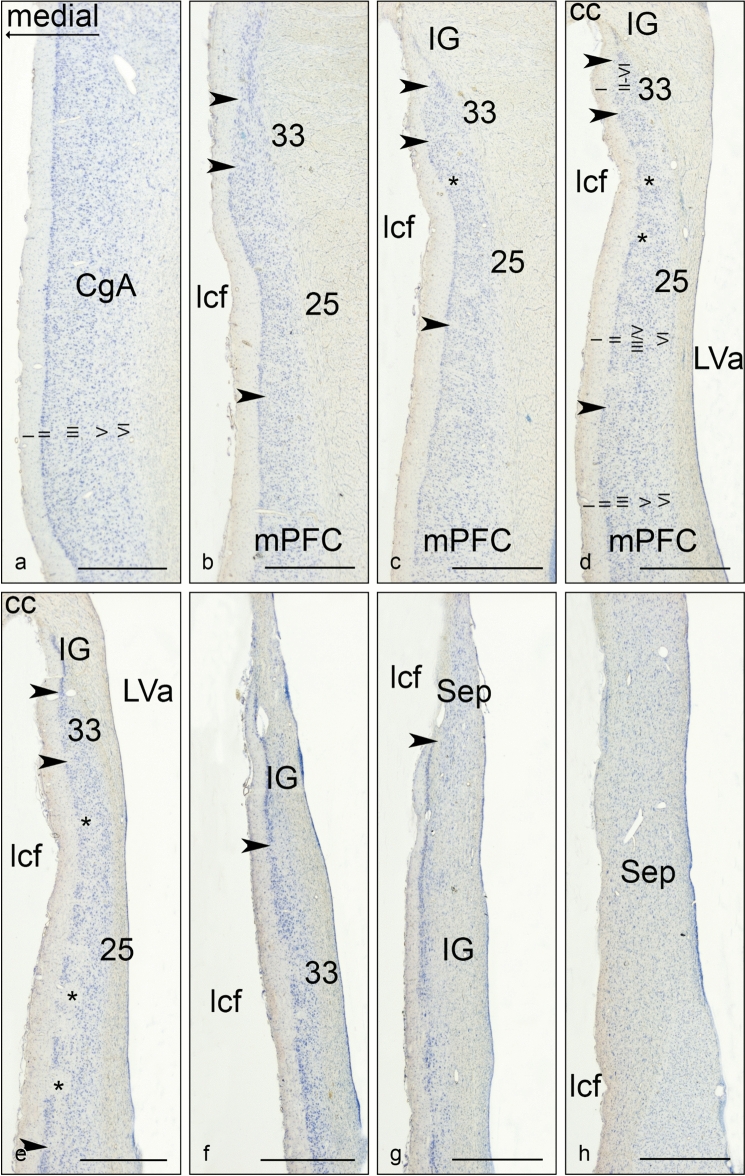
Coronal sections through the sgC. **a**–**h** Corresponds to the lines a-h in Fig. 1b. Arrowheads indicate approximate borders between areas. The relation to the corpus callosum (cc), the longitudinal cerebral fissure (lcf), and the anterior part of the lateral ventricle (LVa) are depicted on **d** and **e**. The cortical layering of the anterior cingulate cortex (CgA) and the BA25 (25) is indicated on **a** and **d**, respectively. Asterisk: cell-poor vertical strips of fiber bundles characteristic of BA25. Scale bar 1000 μm

**Fig. 4:**
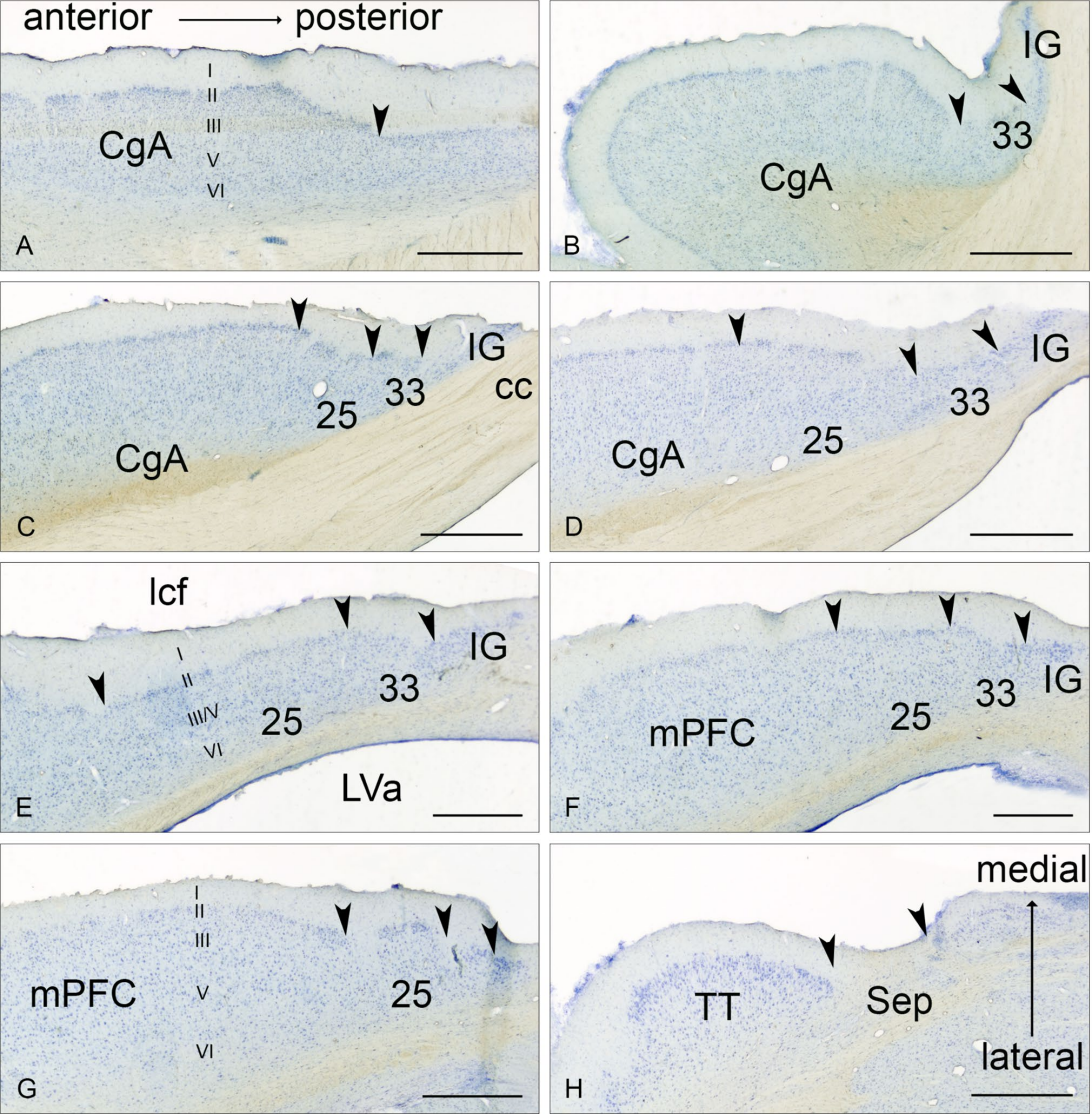
Horizontal sections through the sgC. **a**–**h** Corresponds to lines **a**–**h** in Fig. 1. Arrowheads indicate approximate borders between areas. The position of the corpus callosum (cc) is marked on **c**, while the position of the longitudinal cerebral fissure (lcf) and the anterior part of the lateral ventricle (LVa) is depicted on **e**. The cortical layering of the CgA, BA25 (25), and mPFC is indicated on **a**, **e**, and **g**, respectively. Scale bar 1000 μm

The area identified as the Göttingen minipig sgC is located ventral to the rostral tip of the cc (Figs. 1, 2 and 3). It is irregular-shaped and measures approximately 4.0 × 3.0 × 1.0 mm. It is subdivided into three distinct areas based on cytoarchitectural and laminar differentiation. These areas are in homology with human nomenclature (Brodmann 1909) named area 25 (BA25), area 33 (BA33), and IG. Area 25 is located most anterior in the sgC and is both dorsally and posteriorly aligned by the more undifferentiated area 33 and IG, respectively, which thereby separate area 25 from the cc and the septum (the cell rich part). The sgC is laterally adjoined to the slit-formed anterior part of the lateral ventricle (LVa). The medial–lateral extent of the sgC is thereby restricted to no more than 1 mm at its widest point (Figs. 3c–h, 4e, f).

### Cytoarchitecture of the sgC and adjacent cortical areas

**Fig. 5:**
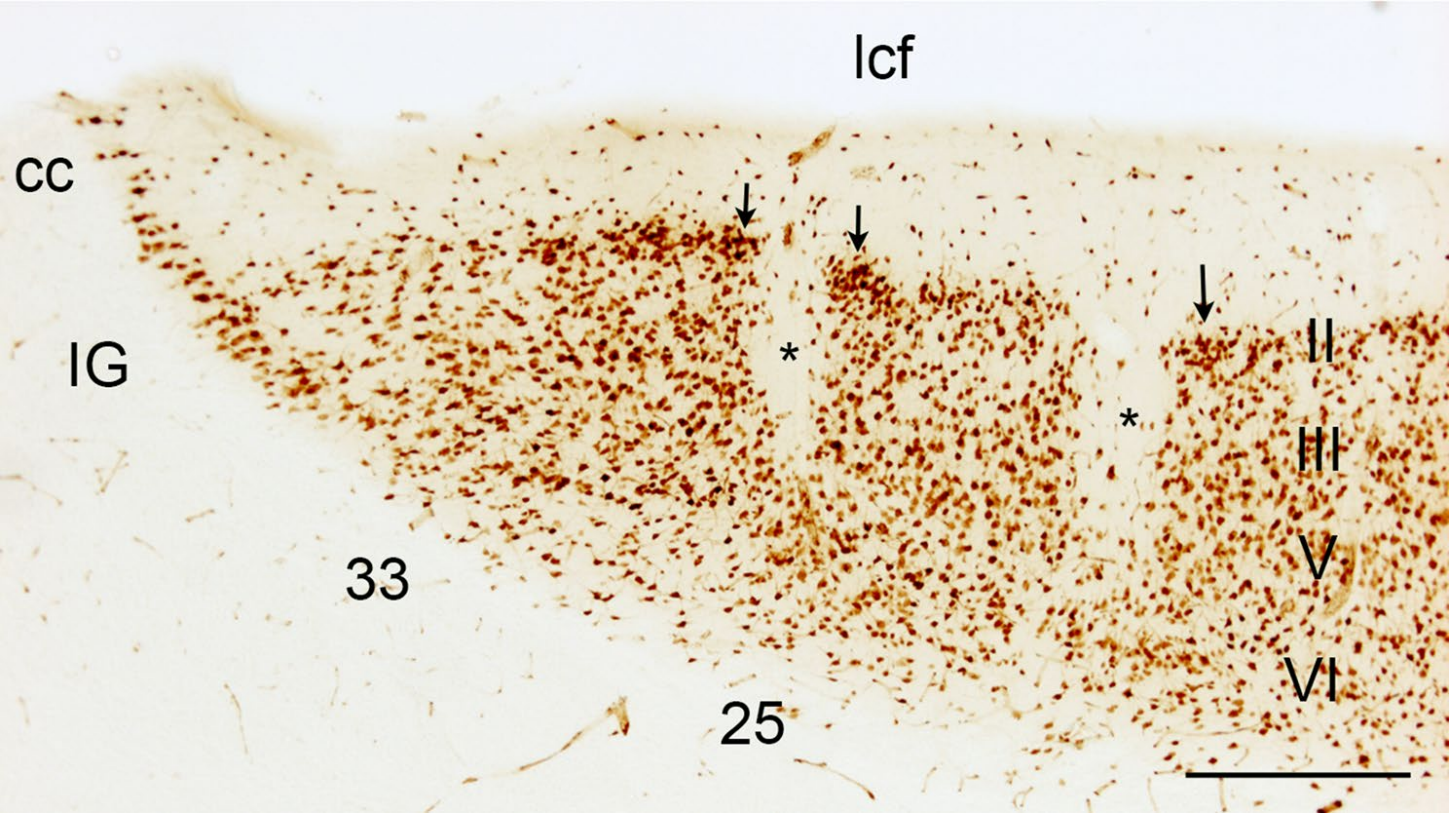
NeuN stained coronal section through the sgC approximately at a position between (**d** and **e** on Figs. 3 and 4. The cortical cytoarchitecture of IG, BA33, and BA25, which constitute the subgenual area, is visible. *Cell poor white matter fiber bundles in BA25, arrow = neuronal clumping in layer II. Scale bar 500 μm

Area 25 (Figs. 3b–e and 4c–g) is a thin agranular cortex, approximately 1 mm thick. Characteristically, cell-poor cortical columns can be found perpendicular to the pial surface segregating small groups of closely arranged neurons in layers II, III and V (Figs. 3c–e and 5). In layer V the cells are of similar size as seen in layer III, while layer VI contains more widely dispersed neurons. The cortex in BA25 lacked a clearly identifiable granular layer (layer IV), making the distinction between layer III and V difficult at some places. However, this distinction becomes more clear when Nissl-stained sections are compared to similar NeuN-stained sections (Figs. 3c–e and 5).

Layer I is thin and cell-poor columns that reach into the deeper layers can be found at regular intervals. Layer II is well-defined and consists of two to three rows of small-sized neurons with sparse perinuclear cytoplasm and circular nuclei. The layer is compact and hence appears to be more intensely stained than the deeper layers. The pyramidal neurons in layer III have almost the same size and staining properties as the medium-sized (15–20 mm) neurons in layer V, and a clear separation between the two layers constituting 12–15 rows of irregularly oriented neurons can therefore be difficult at times. Layer VI is thin and consists of small, evenly stained neurons with smaller somata, and oval nuclei arranged parallel to the surface.

From the coronal and horizontal sections, it is evident that when approaching the cc and septum in the anterior-dorsal plane (Figs. 3b–e and 4c–g), area 25 becomes thinner with increasing cell sparse areas as well as an even more pronounced lack of laminar differentiation.

CgA (Figs. 3a and 4a–d) lies dorsal and anterior to area 25. It is characterized by a larger cortical thickness and densely arranged layer II cells. It is an agranular cortex where layer III and layer V can be more easily segregated as the layer V neurons are slightly larger. Layer VI contains similarly sized but more dispersed cells than layer V.

The mPFC (Figs. 3b–d, 4f, g) lies anterior to the (CgA) and ventral and anterior to BA25. The mPFC contains coarse cells with no apparent small celled granular layer between layer III and V and is accordingly classified as the agranular cortex. Layer II is thin with lightly stained small neurons. Layer III and V have medium-sized and well-dispersed neurons which stain evenly. Layer VI is wide with smaller and more dispersed neurons.

Area 33 wraps around the rostral tip of the cc. Area 33 is i thin and displays a complete lack of laminar differentiation due to diffusely arranged small, lightly stained neurons. These neurons become more intensely stained when approaching the septum (Fig. 3f). Area 33 abuts the IG, which is a neuron dense band of heavily stained small neurons, separating area 33 from the cc dorsally and the septal nuclear area posteriorly.

The septal nuclear area (Figs. 3h and 4h) is delimited anteriorly by the IG. The septal nuclear area is easily recognized by diffusely arranged neurons with oval somata and clear nuclei distributed in a magnocellular medial septal complex and a parvocellular and a magnocellular lateral septal complex (Bjarkam et al. 2017a).

### Neuronal tract tracing

**Fig. 6:**
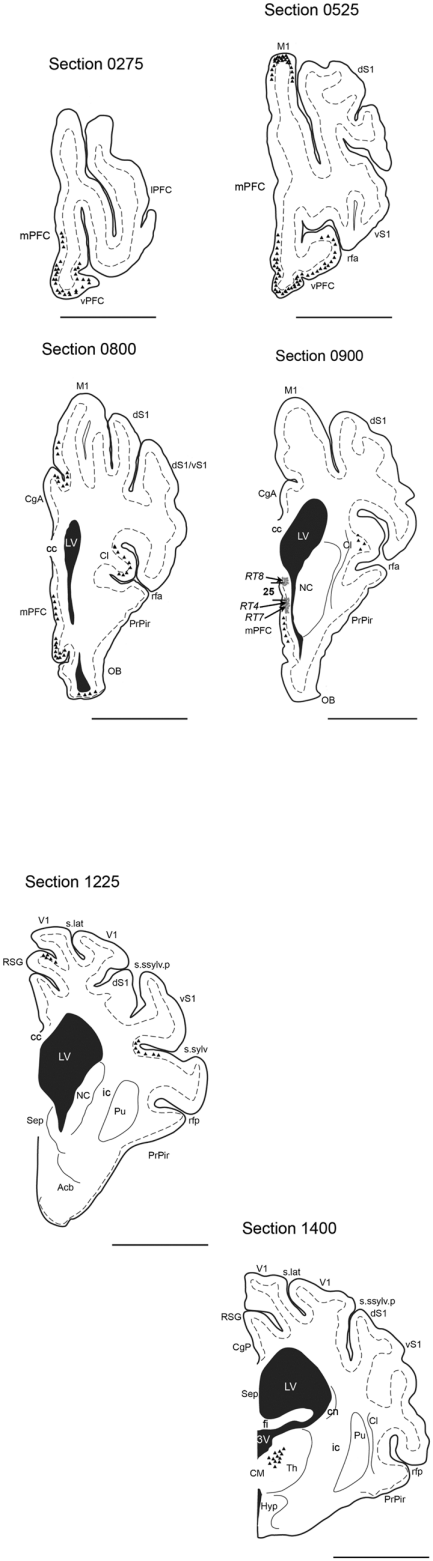

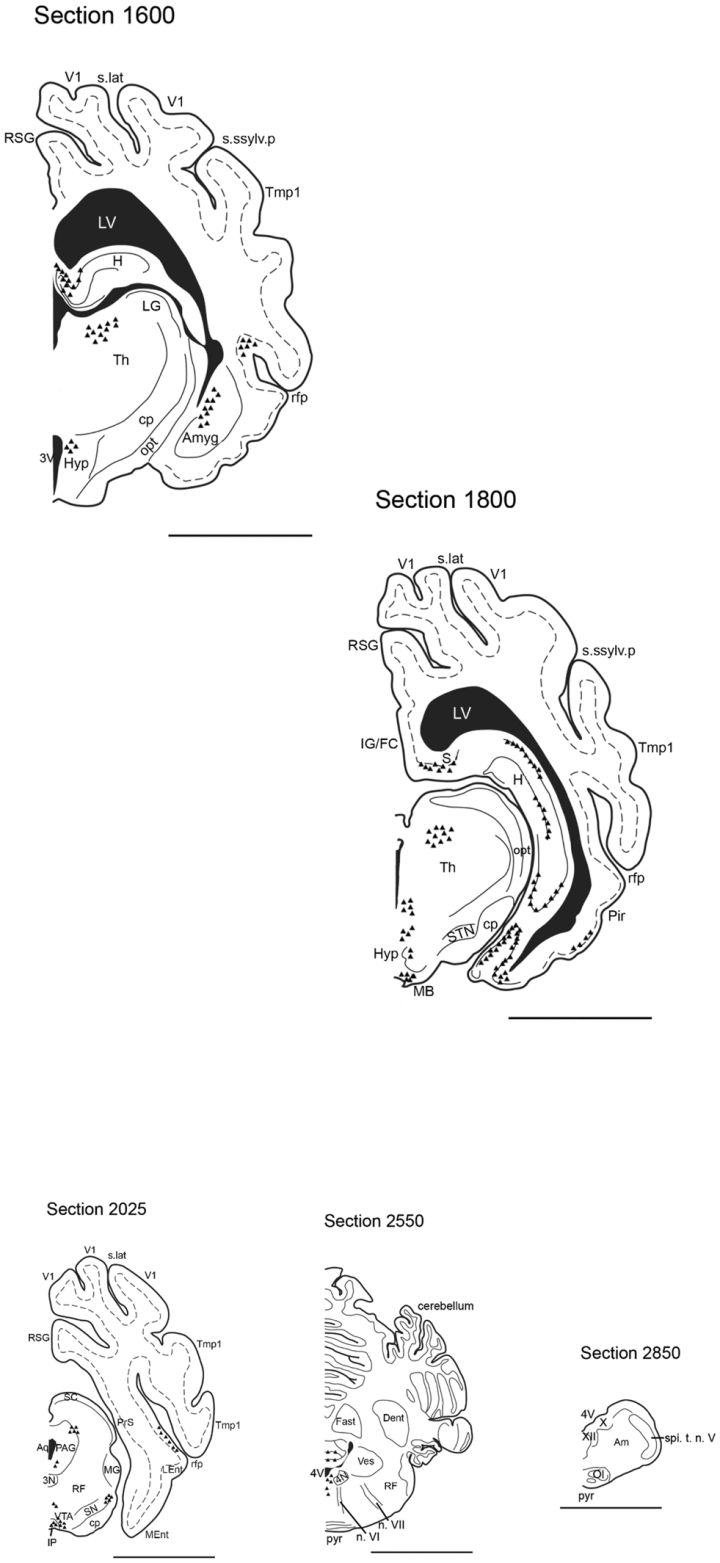
Coronal illustrations depicting the retrograde tracing pattern (triangles) through the Göttingen minipig brain. In Section 0900, the three injection sites are marked RT 4, RT 7, and RT8. The dotted line indicates the cortical layer V where applicable. For abbreviations, see the abbreviation list. Scale bar 1 cm

Due to the limited area size and nearby location to the lateral ventricle and longitudinal cerebral fissure, only 3/8 animals received sgC injections. The location of the trajectories and their retrograde distribution is illustrated in Fig. 6 and presented in the overview in Table 1.

**Table 1:** Summarizes the retro- and anterograde tracing results

Area	Retrograde tracing	Anterograde tracing
*Cortex*		
Medial prefrontal	+ + (b)	+
Ventral prefrontal	+ + + (b)	+ +
Cingulate	+ (b)	+ + + (b)
mPFC/M1 transition	+ + + (b)	+
Insula	+ +	0
Piriform	+ +	+
Prepiriform	+ (b)	–
Entorhinal	+ +	0/ +
Perirhinal	+ +	+
Amygdala	+ +	+
Hippocampus	+ + +	+ +
Subiculum	+ + +	+ +
*Basal ganglia*		
Caudate nucleus	0/ +	+ +
Putamen	0	+
Accumbens nucleus	0	0
*Diencephalon*		
Thalamus	+ + +	+ +
Hypothalamus	+ +	+
*Brainstem*		
Ventral tegmental area	+ +	–
Interpeduncular nucleus	+	–
Substantia nigra	+	–
Periaqueductal grey	+	–
Pontine raphe nuclei	+	–

Tracing results. +  +  + heavy, +  + moderate, + weak, 0 no tracing, – inconclusive, (b) bilateral

### Retrograde tracing

**Fig. 7:**
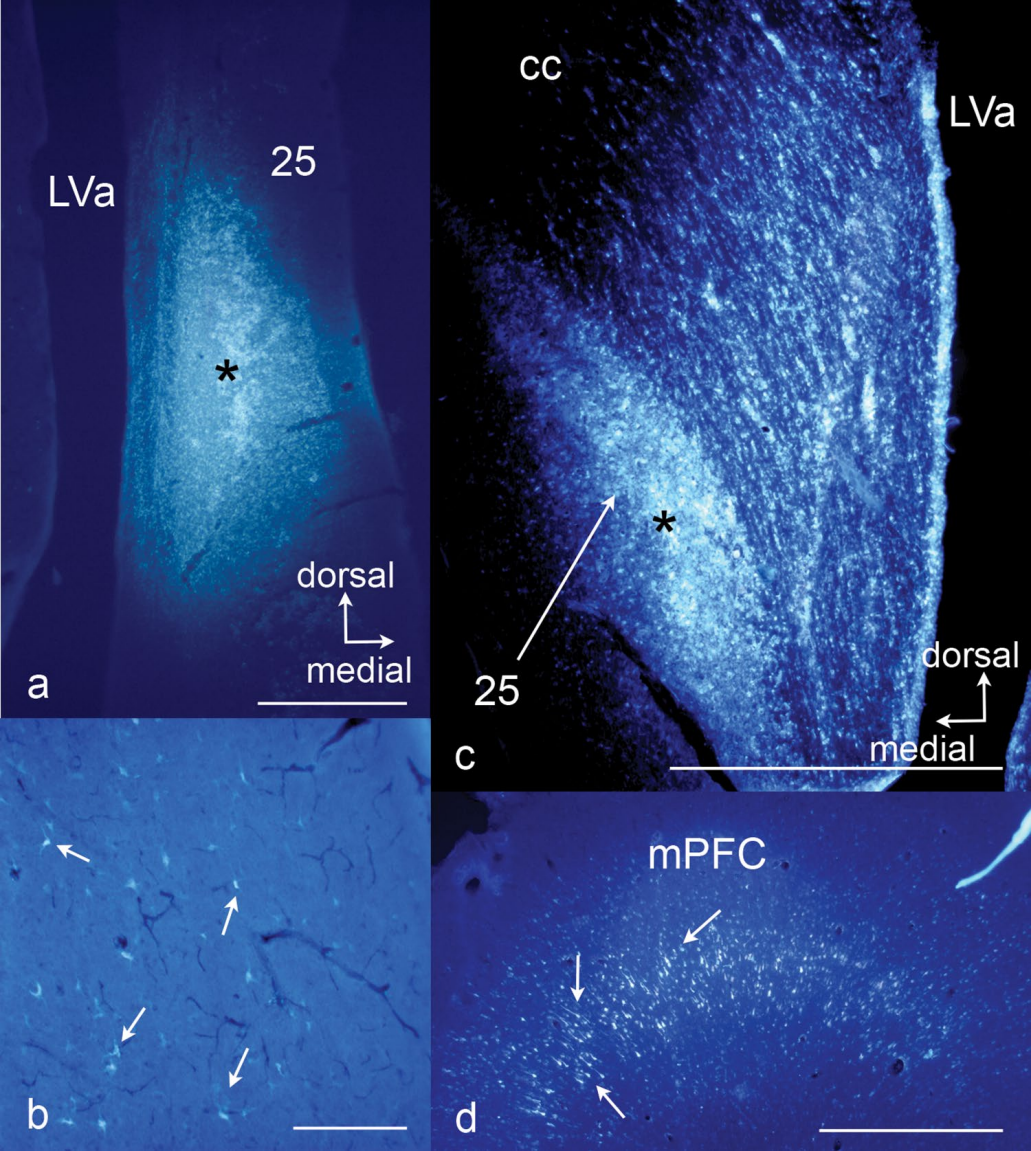
Microphotos showing fluorescent retrograde tracing in the diencephalon (**b**) and mPFC (**d**) after injection (*) with fluorogold in the Göttingen minipig sgC in animal RT4 (**a**) and animal RT8 (**c**). Scale bar (**a**, **c**, **d**) 1 mm. Scale bar (**b**) 250 μm

The FluoroGold tracing was visible as pale, white/yellow labelled fibres and perikarya (Fig. 7). The intensity of the retrograde tracing varied from almost invisible with a few neurons weakly labelled to widespread tracing with numerous neurons almost filled with FG. Schematic illustrations depicting areas with definite tracing are shown in Fig. 6 and schematically listed in Table 1.

Cortical retrograde tracing was seen primarily to the neighbouring ipsilateral ventral and mPFC areas with some contralateral labelling as well. Tracing was most widespread in layer III, but with some labelling of layer V neurons. The densest cortical labelling was found bilaterally in the mPFC (Fig. 7d) with a primarily laminar distribution to layer V. Furthermore, moderate tracing to cortical areas ventral to area 25 was seen bilaterally as well as weak to moderate tracing to the cingulate cortex. Interestingly, ipsilateral retrograde tracing to the insular cortex is clearly seen. Prominent projections were furthermore observed to the medial aspect of the amygdala and the hippocampal formation. Tracing to the hippocampus was seen in the ventral and intermediate parts, but with the heaviest labelling in the dorsal part. The entorhinal cortex (Ent) and subiculum (S) also showed moderate retrograde connections.

### Subcortical retrograde tracing

The basal ganglia received only sparse retrograde tracing. A few neurons in the caudate nucleus (Cd) were labelled, but no tracing was found in the accumbens nucleus (Acb) or the putamen (Pu).

The diencephalon received distinct retrograde tracing primarily to the thalamus (Th). Tracing from anterior and ventral parts of the sgC (RT4 and RT7) resulted in tracing to the anterior and mediodorsal parts of the thalamus, whereas tracing from the posterior and dorsal parts of sgC (RT8) mainly showed tracing to the mediodorsal thalamus. In the hypothalamus (Hyp), weak to moderate tracing was demonstrated in the dorsal and posterior parts.

Brainstem tracing was evident in distinct areas. Heavy labelling appeared bilaterally in the ventral tegmental area (VTA) and ipsilateral in the lateral substantia nigra (SN). Weak projections to the periaqueductal grey (PAG) in the mesencephalon and more caudal to the midline raphe nuclei in pons could also be identified. No retrograde tracing was seen caudal to the pontine area.

### Anterograde tracing

**Fig. 8:**
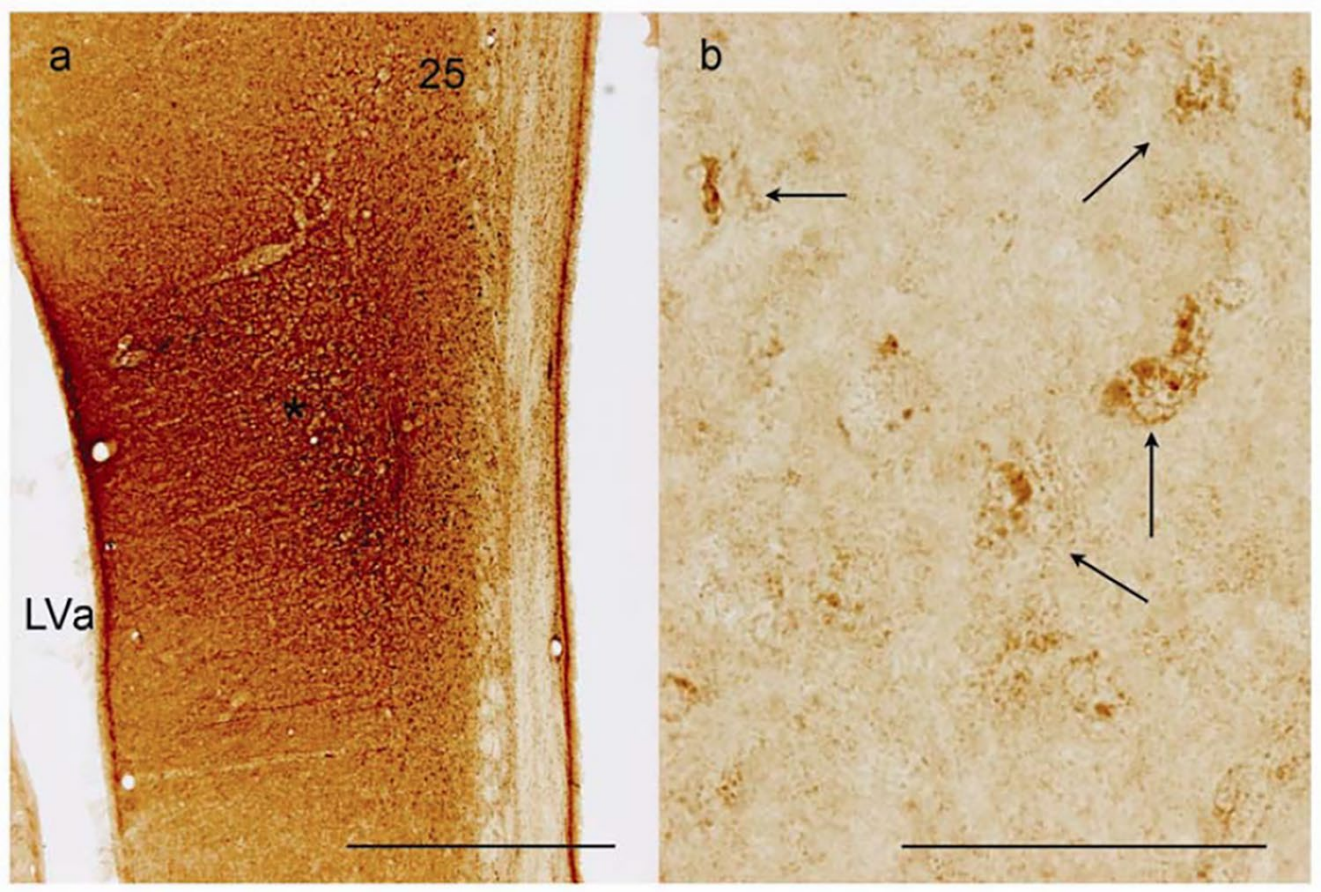
Anterograde tracing visualised with BDA development. **a** Injection site (*) in animal RT4. Scale bar 1 mm. **b** Labelled axon terminals (depicted by arrows) in the thalamus. Scale bar 100 μm

BDA development revealed a moderate distinct brown/black labelling in certain areas, enabling identification of tracer-filled nerve fibers and terminals (Fig. 8).

Cortical projections were observed to the adjoining medial and ventral prefrontal cortex and the cingulate cortex. Dense labelling was observed in the mid-portion of the cingulate gyrus bilaterally as well as in more dorsal and lateral located cortical areas. The hippocampus showed moderate labelling both in the ventral and dorsal parts. Some anterograde tracing was also found in the ventral piriform cortex.

Subcortical projections were less extensively seen. Moderate tracing to the caudate nucleus and the putamen were, however, evident. Just a diffuse weak tracing appeared in the medial aspects of the thalamus and the hypothalamus.

## Discussion

### Anatomy

The sgC has been identified in the Göttingen minipig displaying a similar topography and cytoarchitecture as the human subgenual region (Brodmann 1909). Our findings are in accordance with available publications on the frontal cortex and cingulate gyrus of human, non-human primate, rat and swine (Barbas and Pandya 1989; Carmichael and Price 1994; Jelsing et al. 2006; Vogt et al. 1986; Vogt and Pandya 1987; Vogt et al. 1987; Vogt et al. 1995; Brodmann 1909; Stephan 1951; Walker, 1940; Bjarkam et al. 2017a).

The Göttingen minipig BA25 is agranular cortex characterized by cell-poor cortical columns, located perpendicular to the pial surface, which separate the otherwise cell-rich cortex of layer II and III (Fig. 5). In layer V the cells are more evenly distributed but still of similar size as seen in layer III, while layer VI contains more widely dispersed neurons (Fig. 5). The human area 25 is likewise characterized by such cell-poor columns. It also displays a definite layer II with small aggregation of neurons and a layer VI with small neurons. However, there is a subdivision of layer V into Va and Vb and a more apparent distinction between layer III and V in contrast to what we found in the minipig (Vogt et al. 1995, 2004). Vogt et al. further divided area 25 into a rostral area 25, dissociating from a less differentiated caudal area 25 similar to the area 25 defined by Brodmann. A similar change towards less cortical thickness and layering and more homogeneous cells is noticed when moving posteriorly through the minipig area 25 towards BA33 (Figs. 3, 4, 5). In an extensive investigation on the macaque monkey prefrontal cortex, Walker (1940) identified an area 25 situated in the medial wall of the hemisphere between the anteriorly located areas denominated 10 and 24, and the posteriorly located rostral tip of the corpus callosum. This area 25 has a definite layer IV, contrary to minipig and human. In contrast, macaque’s area 24 is agranular and increasingly simpler in structure when approaching the corpus callosum. Moreover, the neurons in Walker´s area 24 are medium-sized and irregularly arranged. It seems, thus possible, that Walker’s area 25 resembles more well-differentiated granular cortical areas similar to human mPFC, whereas a less organized area more likely to correspond to the human area 25 is included in Walker’s area 24. This would be in concordance with the elaborate work of Carmichael and Price (Carmichael and Price 1994) on the macaque monkey prefrontal cortex using six different staining protocols. They also found an agranular area 25 caudal to a well-defined granular cortex, termed area 10 on the medial hemisphere. Their area 25 lacks clear radial orientation and has a prominent layer V and less dense layer II/III with the inner border of the cortex concluded with a thin layer VI. They give no description of an even less differentiated area caudal to area 25, which could correspond to area 33; however, their illustrations indicate an area termed tenia tectum, which classically is a poorly differentiated cortex although more ventrally located. It remains unelucidated whether this area corresponds to one or both of our areas nominated area 33 and IG, respectively. In the *Macaca mulatta*), area 25 is similarly located as in the *Macaca fascicularis* though, it seems as it extends ventrally into the orbital prefrontal region (Vogt et al. 1987; Barbas and Pandya 1989). The minipig area 25 abuts ventrally the olfactory tubercle and anteriorly to a thicker and more differentiated complex cortex, which we termed the mPFC (Bjarkam et al. 2017a). General features with gradually increasing laminar differentiation and cortical thickness when moving away from the septum and corpus callosum remain applicable in the minipig although the non-human primate area 25 in contrast to our findings seems to have more compact and denser infragranular layers V and VI.

The cytoarchitecture of the swine telencephalon has previously been investigated by Stephan (1951) who unfortunately did not include a separate description of the area termed 25, although his illustrations of the medial surface of the wild boar hemisphere depict such an area ventral to the rostral part of corpus callosum (Stephan 1951). This area is rather large and situated anterior to what is believed to be the septal area. Further rostral, this area abuts an even larger region marked area 8, which is considered granular cortex covering the bulk of the medial frontal surface (Stephan 1951). The cytoarchitecture of the Göttingen minipig prefrontal cortex has likewise been examined previously (Jelsing et al. 2006). In this study, an area denoted infralimbic cortex is recognized ventral to the rostral tip of the corpus callosum. This infralimbic area is not extensively described but does have clusters of cells in layer II as well as vertical cell poor strips. Furthermore, Jelsing et al. (2006), identified a periallocortical area and an IG dorsal to the corpus callosum in accordance with our findings on BA33 and IG. As in our studies (see also Bjarkam et al. 2017a), Jelsing et al. (2006) found the whole of the medial prefrontal area to be agranular, unlike Stephan (1951).

Area 25 is conventionally allocated to the cingulate cortex due to its pregenual localization and agranular appearance and its connectivity with the amygdala (Vogt et al. 2004). Jelsing has likewise included his infralimbic area in the anterior cingulate. We include the sgC in the pericallosal lobe of the Göttingen minipig, which also encompasses cytoarchitectonic distinct anterior cingulate (agranular cortex), posterior cingulate (dysgranular cortex), and retrosplenial cortex (real granular cortex) (Bjarkam et al. 2017a).

### Neuronal Tract Tracing

The small size of the minipig sgC and the anatomical location close to the midsagittal level just below the rather large superior sagittal sinus made stereotaxic targeting difficult. Accordingly, only 3/8 animals had an injection in the sgC (Fig. 6), and some leakage to the ventricular system was subsequently observed in all traced animals.

It was furthermore evident when examining the sections with the fluorescence microscope that the retrograde tracing resulted in nicely labelled neuronal somata and some nerve fibers (Fig. 7), whereas the anterograde labelled terminals were minute and hard to distinguish from background staining (Fig. 8). Hence the anterograde tracing had to be interpreted on BDA-developed sections. The problems regarding the anterograde tracing could be circumvented by the performance of retrograde tracing from areas and structures believed to relate to the area 25. However, the limitations of this time-consuming and resource-demanding method are obvious. By selecting the areas to trace from, one has already opted out some of the possible connections and excluded “unexpected” results. Furthermore, such a procedure would require a large number of animals, and we had already tried to limit the number of used animals by combining the antero- and the posterograde tracing procedure for ethical and economic reasons.

Despite the methodological problems outlined above, we found prominent retrograde connections from several cortical areas. This tracing was mainly confined to the neighbouring ventral and medial prefrontal areas, whereas more sparse connectivity was seen to the cingulate, insular, and perirhinal cortices. Strong projections to the hippocampal regions were observed as well as moderate to weak labelling in the amygdala.

Retrograde connectivity was likewise evident in the anterior, medial and dorsal aspects of the thalamus and posterior hypothalamus. Further caudally afferent connections with the ventral and dorsolateral periaqueductal grey, the ventral tegmental area and the substantia nigra appeared as well as some labelling in the midline raphe nuclei at mesencephalic and pontine levels.

Efferent connectivity included adjacent cortical areas in the cingulate and medial prefrontal cortices, the hippocampus, the caudate nucleus and less prominent tracing to the thalamus and hypothalamus.

### Comparison with other tracing studies

Several studies have examined the connectivity of area 25 and the similar rat infralimbic cortex demonstrating a rich and diverse connectivity pattern (Vogt and Pandya 1987; Barbas and Pandya 1989; Takagishi and Chiba 1991; Condé et al. 1995; An et al. 1998; Ongür et al. 1998; Freedman et al. 2000; Chiba et al. 2001; Vertes 2004; Vogt and Vogt 2004). From these studies, it is clear that area 25 has widespread connections within the frontal cortex, especially the orbitofrontal cortex, as described in our study and reported in different non-human primates and rats (Vogt and Pandya 1987; Barbas and Pandya 1989; Condé et al. 1995). Anterograde tracing with tritiated amino acids injected in different orbital cortices of the rhesus monkey resulted in tracing to area 25 as well as other medial prefrontal cortical areas and adjacent orbitofrontal areas. Injections located in more dorsolateral frontal cortices (areas 46 and 8) did, however, not label terminals in area 25, which is in accordance with the absence of retrograde tracing to dorsolateral frontal cortices in our study (Barbas and Pandya 1989).

As seen in non-human primates and rats (Vogt et al. 1987; Vogt and Pandya 1987; Takagishi and Chiba 1991; Vertes 2004), we found reciprocal connections with the cingulate cortex. Similar to the findings in rat, we found some retrograde labelling in the insular cortex (Condé et al. 1995). This has also been reported in the rhesus monkey (Joyce and Barbas 2018, 2023); furthermore Vogt et al. found that the CgA area 24 in the rhesus monkey received afferents from the insular cortex (Vogt and Pandya 1987).

In the temporal lobe, we found retrograde tracing to the medial aspects of the amygdala. Projections from the amygdala have also been documented in the rhesus monkey and rat primarily located to the basolateral and basomedial nuclei (Vogt and Pandya 1987; Barbas and De Olmos 1990; Condé et al. 1995). We are at present not able to clarify whether these connections in the minipig is reciprocal, however, efferent projections from area 25 to the amygdala is well described in the primate and rat (Hurley et al. 1991; Takagishi and Chiba 1991; Freedman et al. 2000; Chiba et al. 2001; Vertes 2004). Finally, we observed reciprocal connections to the hippocampus and retrograde labelling to the subiculum and the entorhinal cortex, which also is reported in non-human primates (Vogt et al. 1987; Condé et al. 1995). However, hippocampus is not reported as a projection area of area 25 in the Japanese monkey, *Macaca fuscata* (Chiba et al. 2001) or the rat infralimbic area (Hurley et al. 1991). This constitutes a major difference between our study and the latter two studies. One feasible explanation is that our tracer injections are not only confined to the minipig sgC and hence the observed anterograde labelling in the hippocampus could arise due to tracing from adjacent septal areas.

Thalamic projections to area 25 in non-human primates arise primarily in the mediodorsal part of thalamus and midline nuclei and less from more anterior parts of the thalamus (Vogt et al. 1987). Similar findings are seen in the rat, but projections from the anteromedial nucleus in thalamus are also reported (Condé et al. 1995). We found consistent with these results retrogradely labelled neurons in the mediodorsal and anterior aspects of the minipig thalamus. Anterograde tracing from area 25 in the minipig yielded denser labelling in dorsal and midline nuclei and weaker labelling in the mediodorsal aspects. Efferent projections from area 25 in non-human primates and rats to thalamus are found in the paraventricular part of thalamus and the mediodorsal part (Takagishi and Chiba 1991; Freedman et al. 2000; Chiba et al. 2001; Vertes 2004).

Reciprocal connectivity between area 25 and hypothalamus is widespread in the macaque monkey as found by Ongür et al. (Ongür et al. 1998), whereas in the minipig, we found a more restricted retrograde labelling, confined to dorsal and posterior parts of the hypothalamus. Our results are more consistent with data on the rat infralimbic area, in which the hypothalamic anterograde projections are more limited. However, in the rat the projections arise primarily in the lateral hypothalamic area (Conde et al. 1995). The projections from area 25 terminate throughout the macaque hypothalamus, but most densely in the ventromedial nucleus (Ongür et al. 1998; Chiba et al. 2001), whereas in the rat, efferent projections terminate in more lateral and dorsomedial aspects of the hypothalamus. Unfortunately, our anterograde results were technically not conclusive about the efferent projections from the minipig area 25 to hypothalamus.

Anterograde tracing to the caudate nucleus is recognized in our study and is consistent with findings in the studies conducted in both non-human primates (Freedman et al. 2000), where tracing is found in the ventromedial caudate nucleus and dorsomedial caudate, and in rats, where the infralimbic area shows projections to the medial caudate and putamen (Vertes 2004).

Further caudal projections from area 25 in non-human primates are found in different structures in the brainstem, such as the ventral tegmental area, dorsolateral periaqueductal grey, dorsal raphe nuclei and locus coeruleus. Similar projections from the infralimbic area in the rat are found but, in addition, projections continue caudally to the nucleus ambiguous and nucleus of the solitary tract (Hurley et al. 1991; Takagishi and Chiba 1991). Such direct autonomic projections from area 25 in the non-human primates have not been reported (Freedman et al. 2000; Chiba et al. 2001) and thus we would not expect to find such connections in the minipig. However, our study did not provide any results regarding the efferent projections to the minipig’s brainstem; therefore, this cannot be either dismissed or verified. Different areas in the brainstem of the minipig does seem to project to the area 25 such as the ventral tegmental area, the periaqueductal grey, the rostral raphe nuclei and substantia nigra, which is consistent with findings in the rat (Condé et al. 1995) this.

### Functional considerations

Traditionally reciprocal connectivity with the anterior and mediodorsal nucleus of the thalamus has been used to define prefrontal cortical areas and the anterior cingulated cortex, respectively (Devinsky et al. 1995; Vogt et al. 2004; Zilles 2004). In humans the anterior part of the cingulate cortex is classified into the perigenual anterior cingulate cortex (pACC), which comprises the cytoarchitectonic areas termed 32, 24, 25 and the rostral part of area 33 (Vogt et al. 2004). Functionally, the pACC is further subdivided into an area located ventral of the rostrum of the corpus callosum, and the subgenual subregion lying more rostral and ventral to genu corporis callosi comprising the cytoarchitectonic areas 33, 25, 24a and the caudal extreme of area 32 (Vogt et al. 2004).

Comparison of the minipig area 25 to the infralimbic cortex in rats shows some general similarities, but also clear differences (Vogt and Vogt 2004). They both have poor laminar differentiation and thin cortical thickness. Furthermore, the rat layer III is poorly differentiated whereas layer V in the rat is uniform and thick. In the minipig, the discrimination between layer III and V is less obvious, as both layers generally contains evenly sized and dispersed cells (Fig. 5). Importantly although this article does not include behaviour; in rodents, infralimbic cortex is associated with extinction and inhibition of fear responses, whereas in primates, area 25 is not and in fact promotes amygdala activity related to fear and anxiety. (Roberts and Clarke 2019; Wallis et al. 2017).

Our tracing data indicates reciprocal connections between the medial, dorsal and anterior aspects of the thalamus and the subgenual cortex in the minipig, which further strengthens the view that the minipig possesses a cortical area in its subgenual region, homologous to the human area 25. It can furthermore not be disregarded that we found projections from the amygdaloid complex to area 25 in the minipig, which also is found in non-human primates and rats and hence believed to correspond to human connectivity (Vogt et al. 1987, 2004; Vogt and Pandya 1987). Accordingly, it is conceivable that the Göttingen minipig has a homologous subgenual region, which may have similar functional properties. Based on connectional data showing efferent connections from the infralimbic area in the rat to autonomic brainstem nuclei and electrical stimulation of the subgenual subregion eliciting autonomic responses in humans, this region is thought of as a visceromotor control region (Vogt et al. 2004). Considering these data, it is interesting to speculate if this in fact represents the anatomical framework responsible for the autonomic deficits seen in severely depressed individuals.

Interestingly, we also found some rather prominent retrogradely labelled neurons in the ventral tegmental area and less intensively labelling in the pontine and mesencephalic raphe nuclei, which also have been reported in the rat. It is of particular interest that one of the most widely propagated theories concerning the pathophysiology and pharmacological treatment of the major depressive disorder is centred around disturbances in monoaminergic pathways known to originate in part from the identified brain stem structures (Nestler et al. 2002a, b).

## Conclusion

We have identified a sgC in the Göttingen minipig, which based on cytoarchitecture criteria, is segregated into three distinct areas termed area 25, area 33, and IG. Area 25 is, based on comparison with human, non-human primates, and rat studies, believed to be homologous to the human Brodmann area 25 corresponding to area 25 in non-human primates and the infralimbic area in rats. Anterograde and retrograde tracing from the borders of area 25 were furthermore obtained, indicating a largely similar connection pattern as seen in non-human primates and rats. As the identified porcine BA25 area displays a human homolog anatomy (Figs. 1, 2, 3, 4, 5) and is closely connected to major cortical and subcortical limbic key areas (Figs. 6, 7, 8 and Table 1), further experiments examining the mechanisms of human BA25-DBS against depression should be viable in this animal model.

## Data Availability

All material available in the article can be provided on request.
